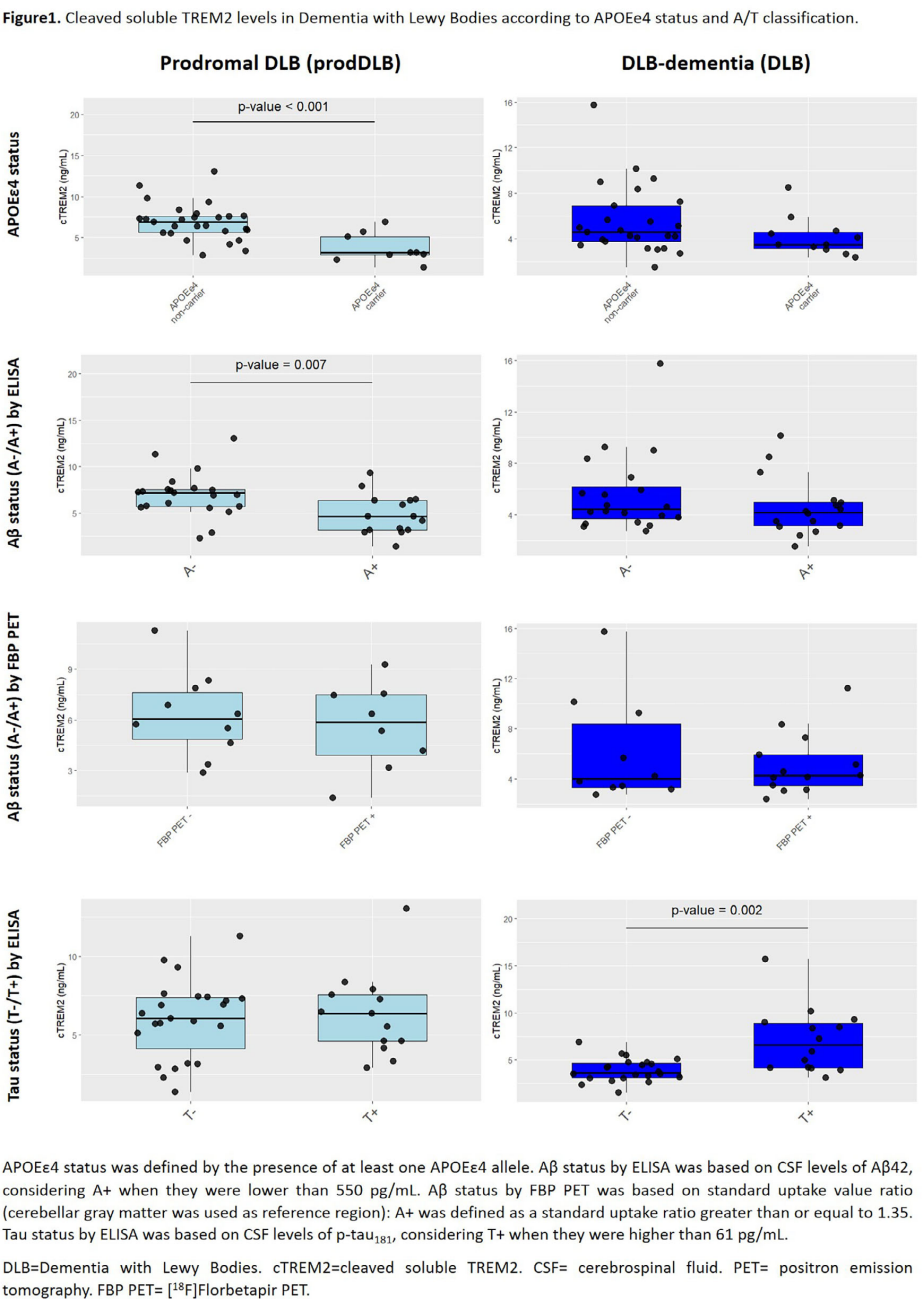# Association between soluble TREM2, APOE genotype, Alzheimer's copathology and disease evolution in dementia with Lewy bodies

**DOI:** 10.1002/alz70855_102529

**Published:** 2025-12-23

**Authors:** Pablo Zaragoza‐Ballester, Briggitte Nuscher, Daniel Alcolea, Álex Fernández‐León, Íñigo Rodríguez‐Baz, Alexandre Bejanin, Elena Vera, Lídia Vaqué‐Alcázar, Isabel Sala, Adolfo Gómez‐Grande, Alberto Lleo, Juan Fortea, Valle Camacho, Christian Haass, Estrella Morenas‐Rodriguez

**Affiliations:** ^1^ Nuclear Medicine Department, Hospital Universitario 12 de Octubre, Madrid, Madrid, Spain; ^2^ Medica School, Universidad Complutense de Madrid, Madrid, Madrid, Spain; ^3^ Neurodegenerative Diaseses Research Group, Hospital Universitario 12 de Octubre (Imas12) Research Institute, Madrid, Madrid, Spain; ^4^ German Center for Neurodegenerative Diseases, Munich, Munich, Germany; ^5^ metabolic Biochemistry, Biomedical Center, Faculty of Medicine, Ludwig‐Maximilians University, Munich, Munich, Germany; ^6^ Sant Pau Memory Unit, Hospital de la Santa Creu i Sant Pau, Institut de Recerca Sant Pau ‐ Universitat Autònoma de Barcelona, Barcelona, Spain; ^7^ CIBERNED, Network Center for Biomedical Research in Neurodegenerative Diseases, National Institute of Health Carlos III, Madrid, Spain; ^8^ Hospital de Sant Pau, Universitat Autònoma de Barcelona, Hospital de la Santa Creu i de Sant Pau, Barcelona, Barcelona, Spain; ^9^ Sant Pau Memory Unit, Hospital de la Santa Creu i Sant Pau ‐ Biomedical Research Institute Sant Pau ‐ Universitat Autònoma de Barcelona, Barcelona, Spain; ^10^ Sant Pau Memory Unit, Hospital de la Santa Creu i Sant Pau, Biomedical Research Institute Sant Pau, Universitat Autònoma de Barcelona, Barcelona, Spain; ^11^ Sant Pau Memory Unit, Hospital de la Santa Creu i Sant Pau ‐ Biomedical Research Institute Sant Pau ‐ Universitat Autònoma de Barcelona, Barcelona, Cataluña, Spain; ^12^ Catalan Foundation for Down Syndrome, Barcelona, Spain; ^13^ Nuclear Medicine Department, Hospital de la Santa Creu i Sant Pau, Barcelona, Barcelona, Spain; ^14^ Munich Cluster of Systems Neurology (SyNergy), Munich, Bavaria, Germany; ^15^ German Center for Neurodegenerative Diseases (DZNE), Munich, Bavaria, Germany; ^16^ Biomedical Center (BMC), Faculty of Medicine, Ludwig‐Maximilians‐Universität München, Munich, Bavaria, Germany; ^17^ Cognitive Disorders Unit, Department of Neurology, Hospital Universitario 12 de Octubre, Madrid, Spain

## Abstract

**Background:**

APOEε4 is a genetic risk factor for both Alzheimer's Disease (AD) and dementia with Lewy bodies (DLB). TREM2‐dependent microglial activation is considered protective in AD and has been proposed to interact with APOE in this context. Since DLB often exhibits Alzheimer's copathology, we investigate the interplay between TREM2 response, APOEε4 carriage and Alzheimer's co‐pathology, and its influence on disease evolution in DLB.

**Method:**

We measured cerebrospinal fluid (CSF) cleaved soluble TREM2 (cTREM2), as a marker of TREM2‐dependent microglial response, core AD biomarkers and determine APOEε4 carriage in 76 DLB patients (prodromal DLB [prodDLB], *n* = 39; DLB‐dementia, *n* = 37). Forty one patients additionally underwent [^18^F]Florbetapir‐PET (FBP‐PET). We quantified cTREM2 by an in‐house MSD‐based immunoassay; core AD biomarkers (Aβ42, t‐tau, *p*‐tau_181_), by ELISA; and FBP‐PET uptake, by standard uptake value ratio (SUVr). We stratified patients according to the A/T classification. Clinical follow‐up (>1 year) was available for 69 patients.

**Result:**

APOEε4 carriers had lower cTREM2 levels compared to non‐carriers in prodDLB (3.72±1.79vs.6.83±2.25ng/mL, *p*‐value=0.0005, Figure 1). Furthermore, APOEε4 carriage itself was associated with lower cTREM2 levels in prodDLB (β(carriers)=‐0.42, *p*‐value=0.025) independently of AD core biomarkers. Conversely, APOEe4 carriage did not impact cTREM2 levels in DLB‐dementia.

cTREM2 levels across A‐/+ and T‐/+ DLB groups are represented in Figure 1. In prodDLB, higher cTREM2 levels were associated with higher Aβ42 (β=0.77, *p*‐value=0.0002) and *p*‐tau_181_ levels (β=0.612, *p*‐value=0.002). In DLB‐dementia, cTREM2 levels were associated only with *p*‐tau_181_ (β=0.58, *p*‐value=0.0001). Additionally, higher cTREM2 levels were associated with lower FBP‐PET SUVr in prodDLB (β=‐1.71, *p*‐value=0.04).

Notably, higher sTREM2 levels at baseline in prodDLB were related to a smaller subsequent longitudinal decrease in MMSE scores (β=1.11, *p*‐value=0.01). No significant relationship was observed between baseline cTREM2 at a DLB‐dementia stage and subsequent cognitive decline (β=‐0.3, *p*‐value=0.5).

**Conclusion:**

APOEε4 carriage attenuates the TREM2‐dependent microglial response in prodromal DLB, as reflected by lower CSF cTREM2 levels. Elevated cTREM2 levels in the prodromal phase are associated with slower cognitive decline, suggesting a protective role of microglial activation during early disease stages. These findings suggest an early modulation of TREM2‐driven microglial response by APOEε4 which influences DLB progression.